# Systemic Delivery of shRNA by AAV9 Provides Highly Efficient Knockdown of Ubiquitously Expressed GFP in Mouse Heart, but Not Liver

**DOI:** 10.1371/journal.pone.0075894

**Published:** 2013-09-24

**Authors:** Bryan A. Piras, Daniel M. O’Connor, Brent A. French

**Affiliations:** 1 Department of Biomedical Engineering, University of Virginia, Charlottesville, Virginia, United States of America; 2 Department of Radiology, University of Virginia, Charlottesville, Virginia, United States of America; 3 Department of Medicine/Cardiovascular Medicine, University of Virginia, Charlottesville, Virginia, United States of America; Justus-Liebig-University Giessen, Germany

## Abstract

AAV9 is a powerful gene delivery vehicle capable of providing long-term gene expression in a variety of cell types, particularly cardiomyocytes. The use of AAV-delivery for RNA interference is an intense area of research, but a comprehensive analysis of knockdown in cardiac and liver tissues after systemic delivery of AAV9 has yet to be reported. We sought to address this question by using AAV9 to deliver a short-hairpin RNA targeting the enhanced green fluorescent protein (GFP) in transgenic mice that constitutively overexpress GFP in all tissues. The expression cassette was initially tested in vitro and we demonstrated a 61% reduction in mRNA and a 90% reduction in GFP protein in dual-transfected 293 cells. Next, the expression cassette was packaged as single-stranded genomes in AAV9 capsids to test cardiac GFP knockdown with several doses ranging from 1.8×10^10^ to 1.8×10^11^ viral genomes per mouse and a dose-dependent response was obtained. We then analyzed GFP expression in both heart and liver after delivery of 4.4×10^11^ viral genomes per mouse. We found that while cardiac knockdown was highly efficient, with a 77% reduction in GFP mRNA and a 71% reduction in protein versus control-treated mice, there was no change in liver expression. This was despite a 4.5-fold greater number of viral genomes in the liver than in the heart. This study demonstrates that single-stranded AAV9 vectors expressing shRNA can be used to achieve highly efficient cardiac-selective knockdown of GFP expression that is sustained for at least 7 weeks after the systemic injection of 8 day old mice, with no change in liver expression and no evidence of liver damage despite high viral genome presence in the liver.

## Introduction

A wide variety of adeno-associated viral (AAV) serotypes have been isolated from multiple species [1]. AAV2 is the most widely studied serotype for direct gene transfer, but it has a low transduction rate and a long lag phase (6 weeks in the heart) prior to maximal gene expression compared to more recently discovered serotypes [2,3]. As a result, these newer serotypes are now being examined for their ability to more efficiently transduce tissues and quickly reach steady-state expression levels. In particular, AAV9 has been shown to provide robust expression in cardiomyocytes, with 358-fold higher luciferase reporter gene expression than AAV2 when injected intravenously into 7 day old mice [4]. In addition, the lag phase for AAV9 is significantly shorter, with expression approaching a steady plateau phase within 3 weeks post-injection in neonatal and adult mice [4].

RNA interference (RNAi) is a powerful technique that provides for the suppression of target genes without the need for homologous recombination or knockout mice. While the knockdown of a gene using RNAi is never complete compared to knockout mice, it is inexpensive and much faster for evaluating the effects of gene knockdown compared to knockout mice. In addition, AAV delivery of RNAi provides temporal control over gene knockdown and is less subject to compensatory mechanisms that may develop over generations of selection in knockout mice. The application of RNAi technology can take many forms, but it is typically implemented within a cell in the form of a 60-70 base-pair short hairpin RNA (shRNA), which is processed into an approximately 20 base pair small interfering RNA through the endogenous microRNA pathway [5]. RNA interference technology is an intense area of research for the development of new therapies, and a number of studies have previously demonstrated the utility of AAV for delivering shRNA in vivo [6,7,8,9].

While AAV9-mediated cardiac-specific transgene overexpression has been demonstrated [4], cardiac-specific knockdown has not. Because most RNA polymerase II promoters, required for tissue-specificity, are not ideal for short transcripts such as shRNA, strictly cardiac-specific shRNA expression is quite challenging. The AAV9 capsid has been shown to be more cardiac-selective than other serotypes, but knockdown expression profiles across multiple tissues after systemic delivery of AAV9 carrying shRNA have not, to our knowledge, been reported. Here, we describe a knockdown system targeting enhanced green fluorescent protein (GFP) in transgenic mice that express GFP under control of the human ubiquitin-C promoter (ubc-GFP) [10]. We then analyzed vector distribution and GFP expression throughout the heart and liver 7 weeks after injection of 8 day old mice and found that, while AAV9 provided highly efficient knockdown in the heart as measured by mRNA and protein analysis, there was no knockdown in the liver despite the presence of 4.5-fold more viral genomes.

## Materials and Methods

### Plasmid Design and In Vitro Validation

**Figure 1:**
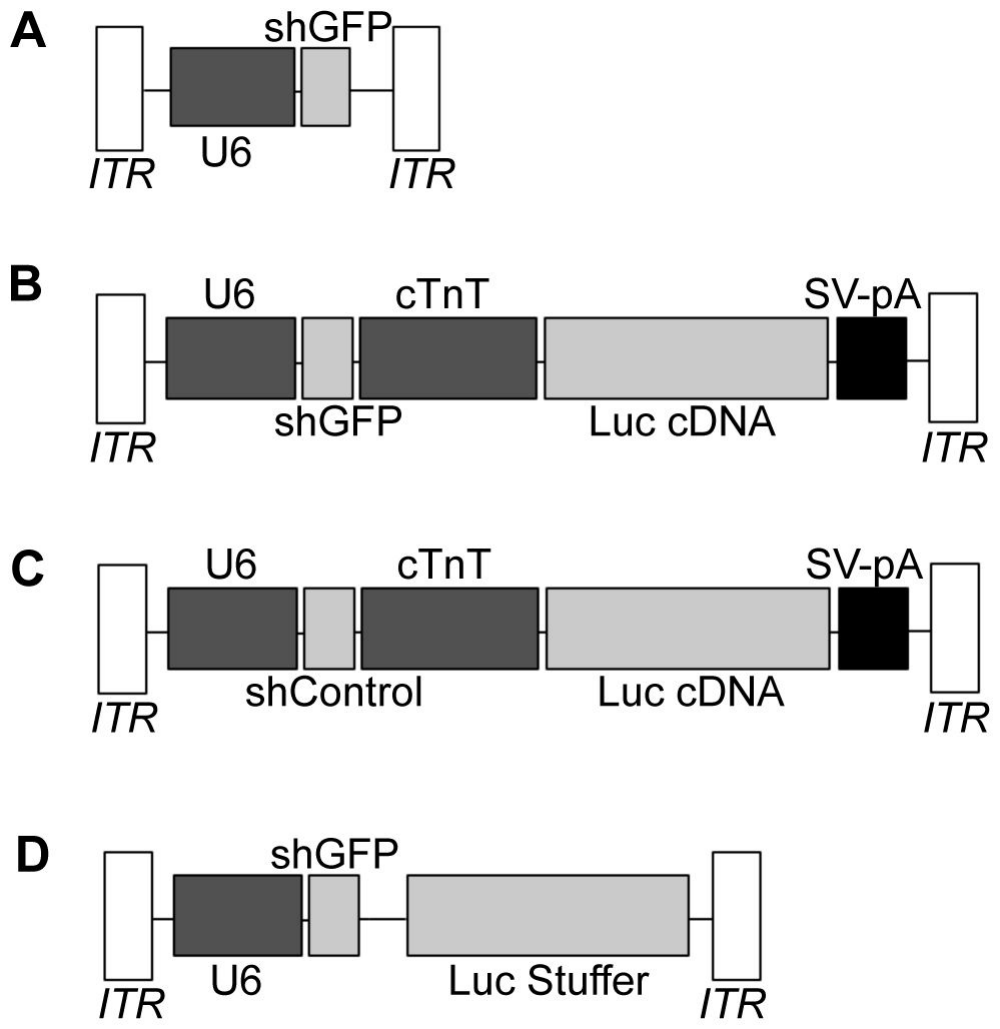
AAV expression cassettes. (A) pAUSiG vector carrying the mouse U6 promoter driving an shRNA against GFP (shGFP) between the two AAV2 ITRs. (B) pAUSiGTL vector (derived from pAUSiG) carrying the cardiac troponin T promoter driving the firefly luciferase reporter (Luc cDNA) with an SV40 polyadenylation signal. (C) A modified version of pAUSiGTL with the shGFP sequence replaced with an shRNA sequence against an off-target gene (shControl). (D) pAUSiGrLuc vector, which contains a luciferase stuffer inserted in reverse orientation without a cTnT promoter.

A knockdown cassette was designed by using PCR to amplify the U6 promoter from mouse genomic DNA and inserting it into a vector containing AAV2 ITRs. A short hairpin RNA containing a target sequence for GFP (shGFP) described by Tiscornia et al. [11] was synthesized and inserted downstream of the U6 promoter. This plasmid, “pAUSiG,” was later modified by inserting a reporter cassette containing firefly luciferase driven by the cardiac troponin T (cTnT) promoter downstream of the U6-shGFP cassette to generate the plasmid “pAUSiGTL.” A control plasmid was made by replacing the shRNA targeting GFP with an shRNA against an off-target gene. To determine whether cardiac-selective knockdown was influenced by the presence of the cTnT promoter, an additional plasmid containing a luciferase stuffer without the cTnT promoter was constructed. These constructs are shown in Figure 1A-D.

Both GFP knockdown plasmids were tested in vitro by cotransfection of the AAV-293 cell line (Agilent Technologies Inc., Clara, CA) via the calcium phosphate method with a plasmid expressing GFP from the cTnT promoter (cTnT-GFP) [12]. Control cells were transfected with cTnT-GFP and a plasmid expressing an off-target shRNA. Three days after transfection, cells were trypsinized and collected for RNA and protein isolation. RNA was isolated with the RNeasy Mini Kit (Qiagen, Inc., Valencia, CA) and reverse transcribed with SuperScript II Reverse Transcriptase (Invitrogen, Carlsbad, CA) for analysis using the Bio-Rad CFX96 Real-Time PCR Detection System (Bio-Rad Laboratories, Hercules, CA). Quantitative PCR was used to assess relative amounts of GFP mRNA present in transfected cells using the comparative C_T_ method, with expression normalized by GAPDH. Western analysis was used to evaluate GFP protein content by homogenizing cells in buffer containing 50 mM Tris-HCl, 2 mM each of EDTA and EGTA, 0.3% Triton-X 100, and a protease inhibitor cocktail (Thermo Scientific, Rockford, IL). Total protein was quantified with the Bio-Rad DC Protein Assay, and blots were probed with goat anti-GFP (BA-0702, Vector Laboratories, Inc., Burlingame, CA) at a 1:3000 dilution prior to the application of a biotinylated secondary antibody with a chemiluminescent substrate for quantification.

### AAV Vector Production

Vector genomes with AAV2 ITR sequences were cross-packaged into AAV9 capsids via triple transfection of AAV-293 cells, then purified by ammonium sulfate fractionation and iodixanol gradient centrifugation. Titers of the AAV vectors [viral genomes (vg)/ml] were determined by qPCR. The following primers were used for amplifying the mouse U6 promoter: 5’-TCGCACAGACTTGTGGGAGAA-3’ (forward) and 5’- CGCACATTAAGCCTCTATAGTTACTAGG-3’ (reverse). Known copy numbers (10^5^–10^9^) of plasmids carrying the corresponding expression cassettes were used to construct standard curves for quantification.

### Animal Procedures

The animal protocol used in this study was approved by the University of Virginia Institutional Animal Care and Use Committee (Protocol Number: 2802) and strictly conformed to the “Guide for the Care and Use of Laboratory Animals” (NIH Publication 85-23, revised 1985). ubc-GFP mice were maintained on a 12/12 hr light/dark cycle at 24°C and 60% humidity.

### Vector Administration

To deliver virus, 8 day old ubc-GFP mice were anesthetized with 1-1.2% isoflurane in oxygen and a needle was inserted through the thoracic wall into the left ventricular chamber. Blood was withdrawn from the ventricular chamber to confirm systemic delivery, after which viral solution (50-100 µl containing 1.8×10^10^ to 1.8×10^11^ vg for the dose ranging study or 4.4×10^11^ vg for other studies) was slowly injected.

### Bioluminescence Imaging

Luciferase expression was assessed in live mice using an in vivo bioluminescence imaging system (IVIS 100, Caliper Life Sciences, Hopkinton, MA) as described previously [2,13,14]. Briefly, mice were anesthetized with isoflurane and injected with 150 µL of 30 mg/mL D-luciferin (Gold Biotechnologies, Inc., St. Louis, MO) intraperitoneally. Images were collected 10-15 minutes after substrate injection.

### Fluorescence Microscopy

For fluorescence microscopy, heart and liver tissues obtained 7 weeks after vector injection were excised and fixed for one hour at room temperature in 4% PFA, rinsed in PBS, and incubated overnight at 4°C in 30% sucrose in PBS before embedding in OCT. Six micron cryosections were cut from each tissue and analyzed with an Olympus BX-41 Microscope (Olympus, America, Inc., Center Valley, PA) with a Retiga-2000R camera (QImaging, Surrey, BC). Further imaging was performed with an Olympus IX81 inverted microscope with 10× UPlanFLN 0.30 NA objective, Orca-AG CCD camera (Hamamatsu, Bridgewater, NJ), automated stage (Prior Scientific, Rockland, MA), and IPLab software (Scanalytics, Fairfax, VA).

### Histology

Liver tissue prepared for fluorescence microscopy was also assessed for potential damage caused by expression of shRNA. Six micron liver sections were stained with hematoxylin and eosin and photographed using an Olympus BX-51 high magnification microscope with attached Olympus DP70 digital camera.

### Determination of AAV Vector Genome Copy Number

Total genomic DNA was isolated from mouse hearts with the Qiagen AllPrep DNA/RNA system (Qiagen Inc., Valencia, CA). AAV vector genome copy numbers were determined by qPCR using the Bio-Rad iTaq Universal SYBR Green Supermix PCR kit (Bio-Rad Laboratories, Hercules, CA) and a Bio-Rad CFX Connect real-time system. The following primers were used to amplify luciferase: 5’- AAGATTCAAAGTGCGCTGCTGGTG-3’ (forward) and 5’- TTGCCTGATACCTGGCAGATGGAA-3’ (reverse). Known copy numbers (10^5^–10^9^) of a plasmid containing the luciferase gene were used to construct the standard curve. Results are expressed as the number of vector copy numbers per μg of genomic DNA.

### mRNA Analysis

After hearts and livers were harvested, one-third of each was frozen in RNAlater (Qiagen Inc.) for storage. Tissues were subsequently processed with the Qiagen AllPrep DNA/RNA system, after which mRNA was converted to cDNA using the Bio-Rad iScript cDNA Synthesis Kit before qPCR analysis with the Bio-Rad iTaq Universal SYBR Green Supermix. The following primers were used for amplifying GFP: 5’- TGACCCTGAAGTTCATCTGCACCA-3’ (forward) and 5’- TCTTGTAGTTGCCGTCGTCCTTGA-3’ (reverse); and, for normalization, GAPDH: 5’- TCAACAGCAACTCCCACTCTTCCA-3’ (forward) and 5’- ACCCTGTTGCTGTAGCCGTATTCA-3’ (reverse).

### Western Analysis

Immunoblots were used to evaluate GFP content in heart and liver by homogenizing tissues in buffer containing 50 mM Tris-HCl, 2 mM each of EDTA and EGTA, 0.3% Triton-X 100, and a protease inhibitor cocktail. Protein was quantified with the Bio-Rad DC Protein Assay, and goat anti-GFP (BA-0702, Vector Laboratories, Inc., Burlingame, CA) was applied at a 1:3000 dilution prior to incubation with a fluorescent secondary antibody and detection with the Odyssey Imaging System (LI-COR Biosciences, Lincoln, NE). Signals from an anti-actin antibody (A2103, Sigma-Aldrich, St. Louis, MO) or an anti-GAPDH antibody (600-401-A33; Rockland Immunochemicals Inc., Gilbertsville, PA) were used as loading controls.

### Statistical Analysis

Data are expressed as mean ± SEM where appropriate. Results from the dose-dependent study were analyzed by the nonparametric Kruskal-Wallis test. All other comparisons were analyzed with Student’s t-test. P<0.05 was considered significant in all comparisons.

## Results

### Efficient In Vitro Knockdown of GFP

**Figure 2:**
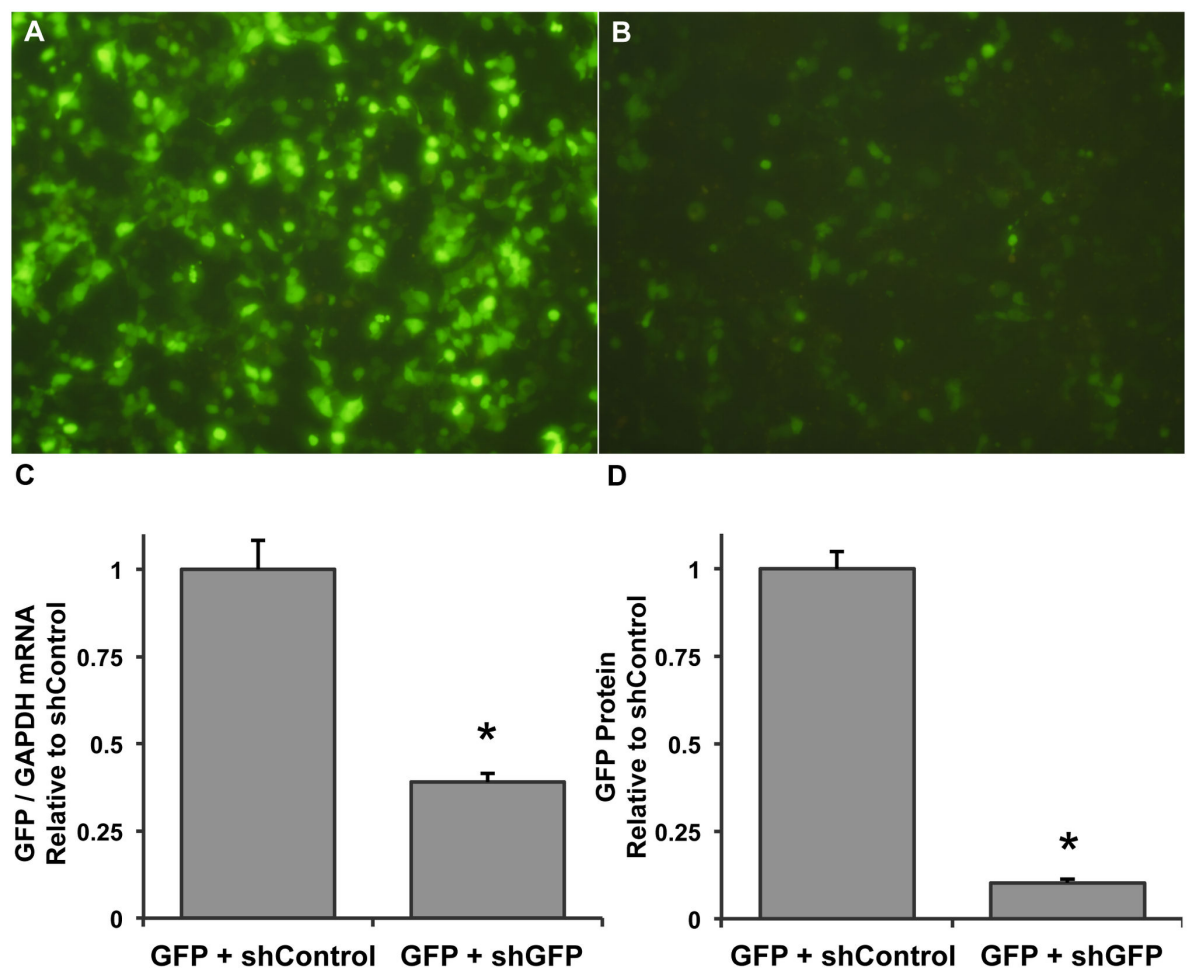
Efficient shRNA-mediated knockdown of GFP in vitro. (A) AAV-293 cells dual-transfected with a plasmid expressing GFP from the cardiac troponin T promoter and a plasmid expressing a negative control shRNA from the mouse U6 promoter (shControl). (B) Cells transfected similarly but with the pAUSiGTL plasmid expressing shGFP. Cells were imaged 3 days post-transfection. (C) GFP mRNA from transfected cells harvested 3 days post-transfection. GAPDH was used to normalize GFP expression, which was found to be reduced by 61% compared to the shControl-treated group (n=3/group, p<0.01). (D) GFP protein expressed relative to the shControl-treated group showed a 91% reduction in the shGFP group (n=3/group, p<0.001).

The shGFP expression cassette was tested in vitro by cotransfection via the calcium phosphate method with a plasmid expressing GFP from the cTnT promoter. Control cells were transfected with cTnT-GFP and a plasmid expressing an off-target shRNA. Efficient knockdown of GFP was achieved 3 days after cotransfection (Figure 2A-B). Results from qPCR indicated a 61% reduction in GFP transcript (Figure 2C, n=3/group, p<0.01 vs. control), while Western blot analysis revealed a 90% reduction of GFP protein compared to the off-target shRNA control (Figure 2D, n=3/group, p<0.001).

### shRNA-Mediated Knockdown in Heart is Dose-Dependent

**Figure 3:**
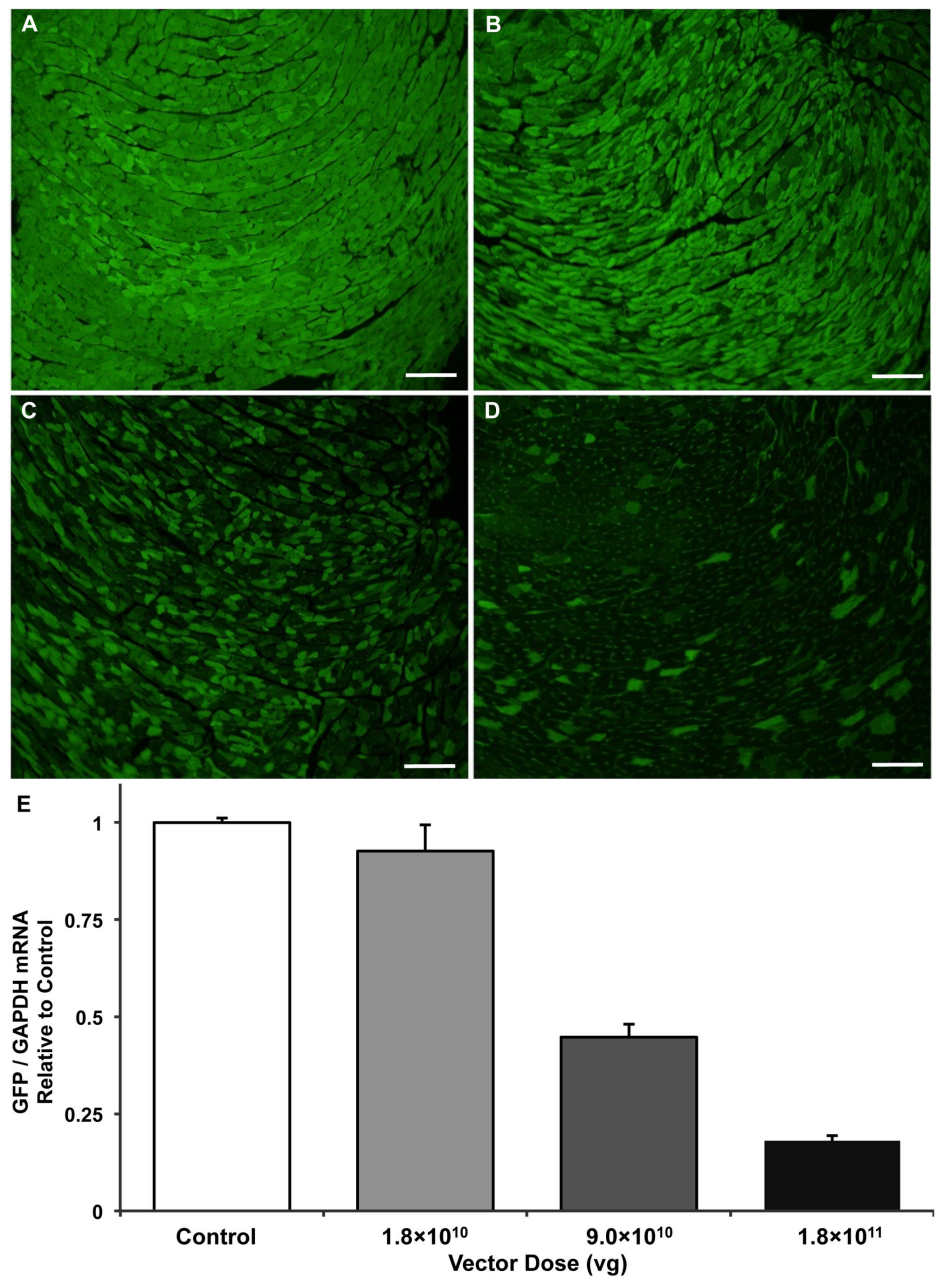
shRNA-mediated knockdown in the heart is dose-dependent. Fluorescent tissue sections showing GFP expression in ubc-GFP mice 7 weeks after: (A) being treated as uninjected controls (n=2) or (B)-(D) injection with 1.8×10^10^ (n=3), 9.0×10^10^ (n=3), or 1.8×10^11^ (n=2) viral genomes per mouse, respectively. Scale bars are equal to 50 microns. (E) GFP mRNA from harvested cardiac tissue, normalized by GAPDH and expressed relative to the uninjected control group. qPCR analysis of mRNA demonstrated a 55% reduction in GFP mRNA with the medium dose of 9×10^10^ vg and an 82% reduction in transcript with the high dose of 1.8×10^11^ vg compared to the control group (p=0.052).

To test the effect of dose on GFP knockdown, 3 different doses of the pAUSiG vector packaged in AAV9 were administered to ubc-GFP mice: 1.8×10^10^ vg (n=3), 9×10^10^ vg (n=3), and 1.8×10^11^ vg (n=2). Control mice (n=2) were left uninjected. Seven weeks after injection, mice were euthanized and their hearts were collected for fluorescence microscopy and mRNA analysis. Figure 3A-D shows fluorescence micrographs of hearts from control mice (A) and mice that received 1.8×10^10^ vg (B), 9×10^10^ vg (C) and 1.8×10^11^ vg (D), demonstrating a decrease in fluorescent cells with increasing dose of AAV9. These results were confirmed with qPCR analysis of mRNA, which demonstrated a 55% reduction in mRNA with the medium dose of 9×10^10^ vg and an 82% reduction in transcript with 1.8×10^11^ vg compared to the control group (Figure 3E, p=0.052).

### Luciferase Provides an Independent Confirmation of Gene Expression When Placed Downstream of a Knockdown Cassette

**Figure 4:**
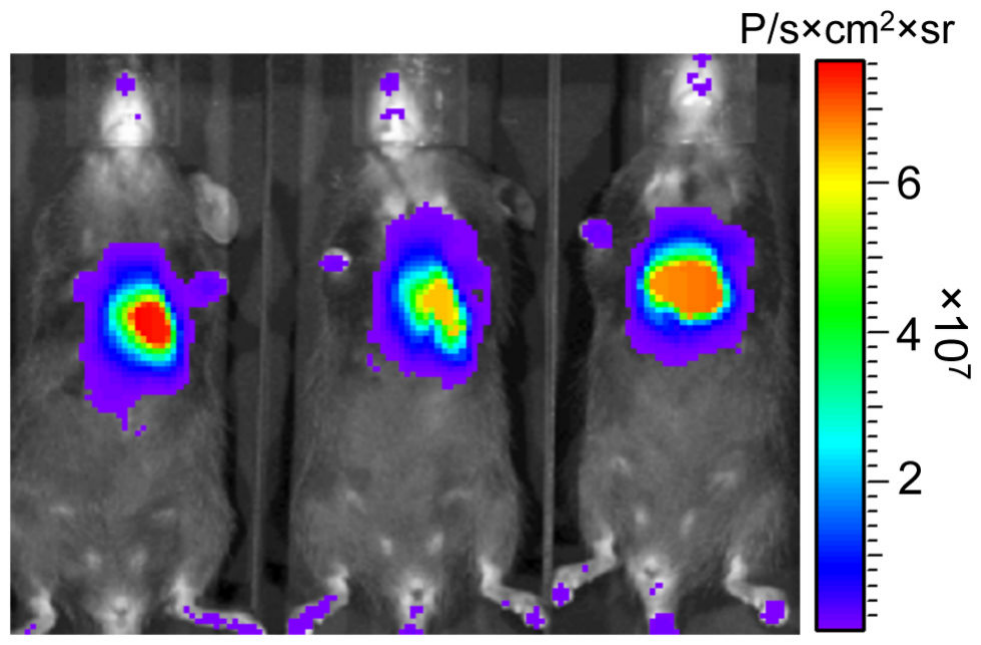
Luciferase provides an independent confirmation of gene expression when placed downstream of a knockdown cassette. Further experiments used the pAUSiGTL vector packaged in AAV9 to provide an independent indicator of gene expression with the luciferase reporter. Mice imaged by bioluminescence 7 weeks after injection had cardiac restricted expression of luciferase as a result of the cardiac-specific cTnT promoter. These results provided confirmation of viral delivery and expression in live mice prior to tissue-level analysis of gene knockdown.

For all remaining experiments, the pAUSiGTL vector was packaged into AAV9 to provide an independent confirmation of gene transfer via the luciferase reporter gene. Seven mice were injected with 4.4×10^11^ vg AAV9-pAUSiGTL, while an additional 7 mice were injected with 4.4×10^11^ vg of a similar vector containing an off-target shRNA as a control (shControl). Mice imaged 7 weeks after injection demonstrated cardiac-restricted expression of luciferase from the cardiac-specific cTnT promoter (Figure 4). These results provided confirmation of viral delivery and gene expression in live mice prior to tissue-level analysis of gene knockdown.

### Highly Efficient Knockdown of GFP in Heart but Not Liver

**Figure 5:**
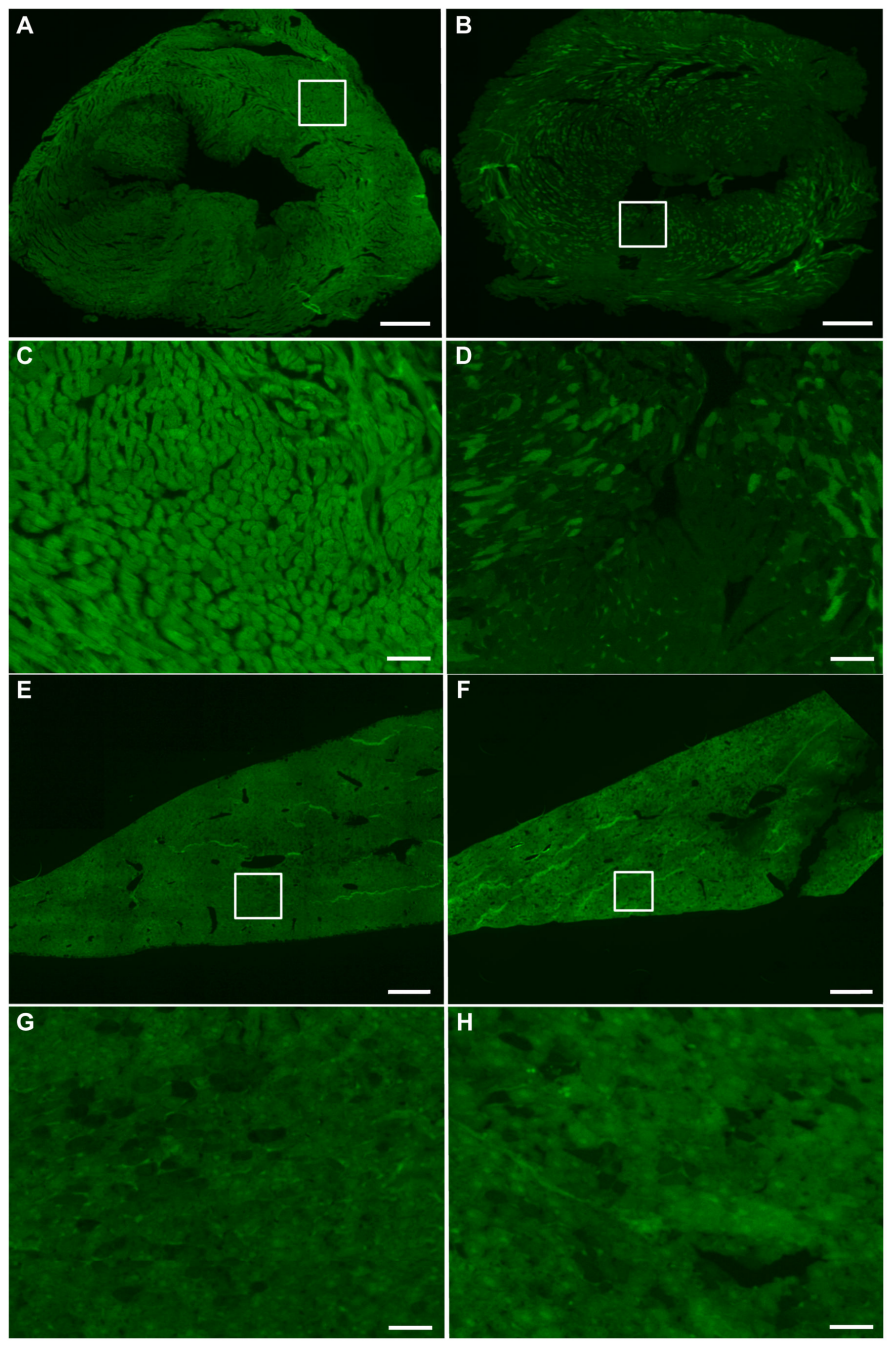
Fluorescent sections from heart and liver show highly efficient cardiac knockdown and no liver knockdown. Mice were injected with AAV9 capsids containing the single-stranded pAUSiGTL vector expressing shGFP or AAV9 containing an off-target shRNA. Tissues were harvested 7 weeks after injection. (A) and (E) show heart and liver, respectively, treated with the off-target shRNA (scale bars = 500 µm), while (C) and (G) show the same sections magnified in the area of the white boxes (scale bars = 50 µm). (B) and (F) show heart and liver, respectively, treated with shGFP (scale bars = 500 µm), while (D) and (H) show the same sections magnified in the area of the white box (scale bars = 50 µm).

**Figure 6:**
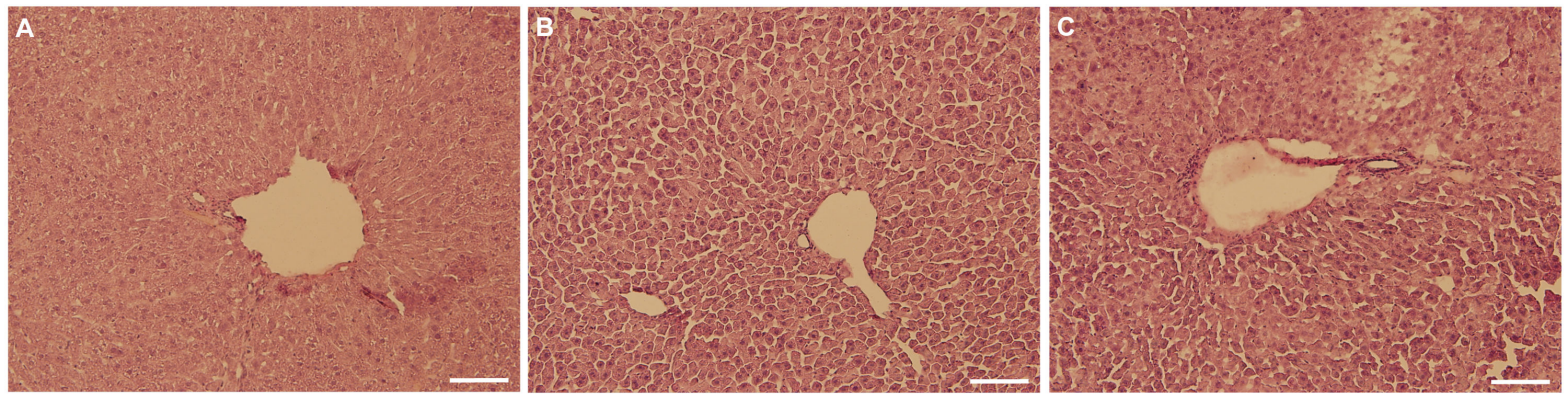
H&E staining shows no histological evidence of liver damage. Liver sections photographed after staining with hematoxylin and eosin show no signs of liver damage when comparing: (A) liver treated with a control AAV not expressing shRNA to (B) shControl and (C) shGFP treated liver. Scale bars are equal to 100 µm.

After imaging, mice were euthanized and their hearts and livers were collected for analysis by fluorescence microscopy, as well as for analysis of AAV genomes, GFP mRNA and GFP protein. Six micron cryosections of heart and liver tissue revealed a large difference between the number of fluorescing cardiomyocytes treated with shControl and shGFP (Figure 5A& C and B& D, respectively), while there was no appreciable difference in fluorescence between groups in the liver (Figure 5E& G and F& H). While shControl-treated ubc-GFP mouse hearts were nearly homogenous in fluorescence, the majority of cardiomyocytes in shGFP-treated mice did not exhibit fluorescence levels above background. Liver sections from both groups appeared slightly less homogenous, with no apparent difference between treatment groups. Liver sections were also stained with hematoxylin and eosin to detect potential liver tissue damage due to shRNA production as reported previously [9]. Representative H&E stained liver sections after treatment with a control AAV expressing Cre recombinase from a periostin promoter (Figure 6A), shControl AAV (Figure 6B) and shGFP AAV (Figure 6C) showed no histological evidence of damage.

**Figure 7:**
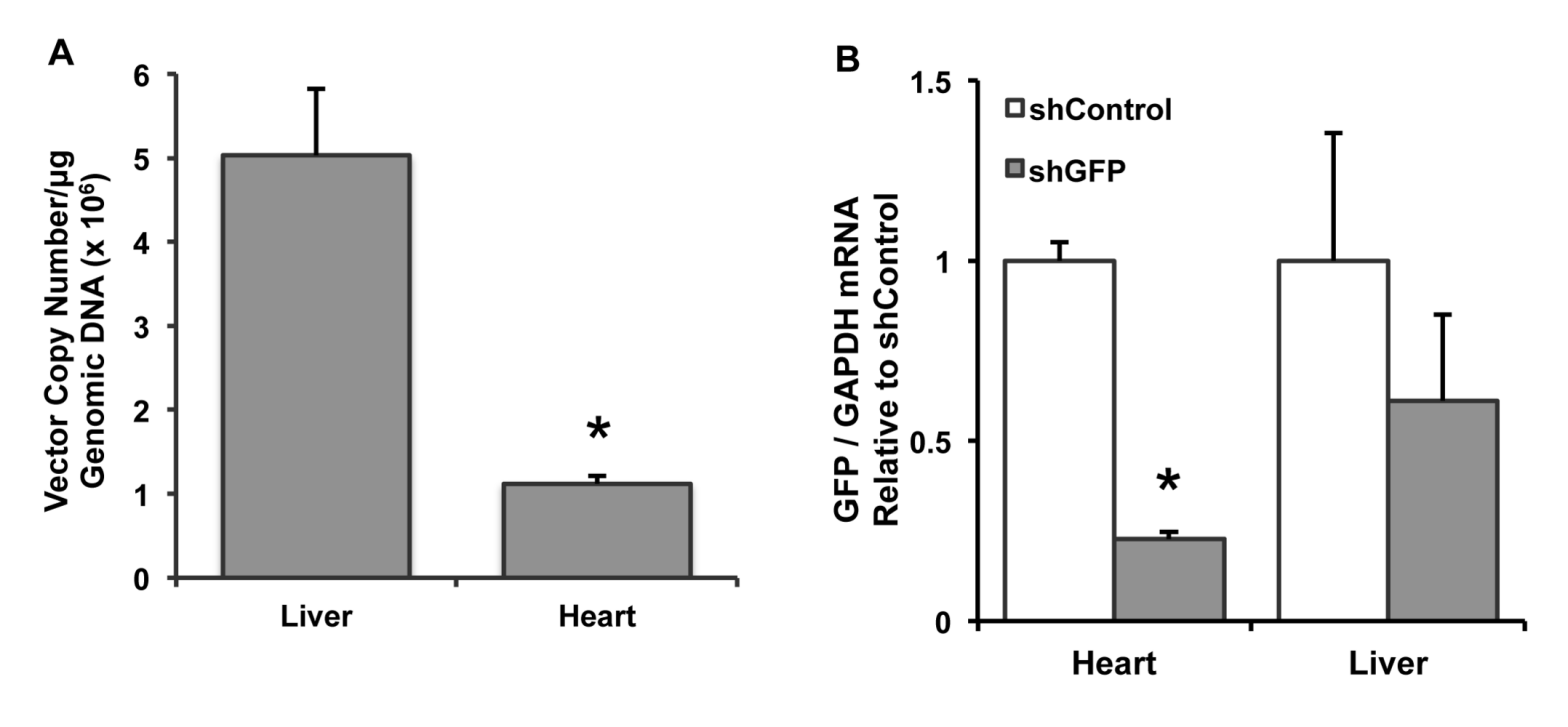
Vector genomes and GFP mRNA in heart and liver. (A) To determine viral distribution within the primary target tissues, genomic DNA (gDNA) was isolated from heart and liver and analyzed by qPCR to find average vector copy numbers per microgram of gDNA. Liver had approximately 4.5-fold more vector genomes than the heart (5.0 ×10^6^ copies/μg gDNA versus 1.1×10^6^ copies/μg gDNA, respectively; n=6/group, p<0.001). (B) mRNA was also isolated from heart and liver, and GFP transcript levels were normalized by GAPDH. The shGFP group showed a 77% reduction in cardiac GFP mRNA compared to the shControl group (n=7/group, p<0.0001), and while the shGFP group showed a 39% decrease in mRNA in liver, the difference was not statistically significant (n=7/group, p=0.38).

To assess the distribution of viral genomes between tissues, genomic DNA (gDNA) was isolated from heart and liver and analyzed by qPCR to determine mean vector copy numbers per microgram of gDNA. Figure 7A shows that liver had approximately 4.5-fold more vector genomes than the heart (5.0×10^6^ copies/μg gDNA versus 1.1×10^6^ copies/μg gDNA, respectively; n=6/group, p<0.001). In addition to gDNA, mRNA was also isolated from heart and liver. After conversion to cDNA, qPCR was performed to assess GFP transcript levels normalized by GAPDH transcripts in each tissue. Figure 7B shows the expression of GFP/GAPDH mRNA relative to the shControl group for heart and liver. The shGFP group in heart showed a 77% reduction in GFP mRNA compared to the shControl group (n=7/group, p<0.0001). While the shGFP group showed a 39% decrease in GFP mRNA in liver, this difference was not statistically significant (n=7/group, p=0.38). These results show that despite increased prevalence of viral genomes in the liver, greater knockdown of GFP was achieved in the heart.

**Figure 8:**
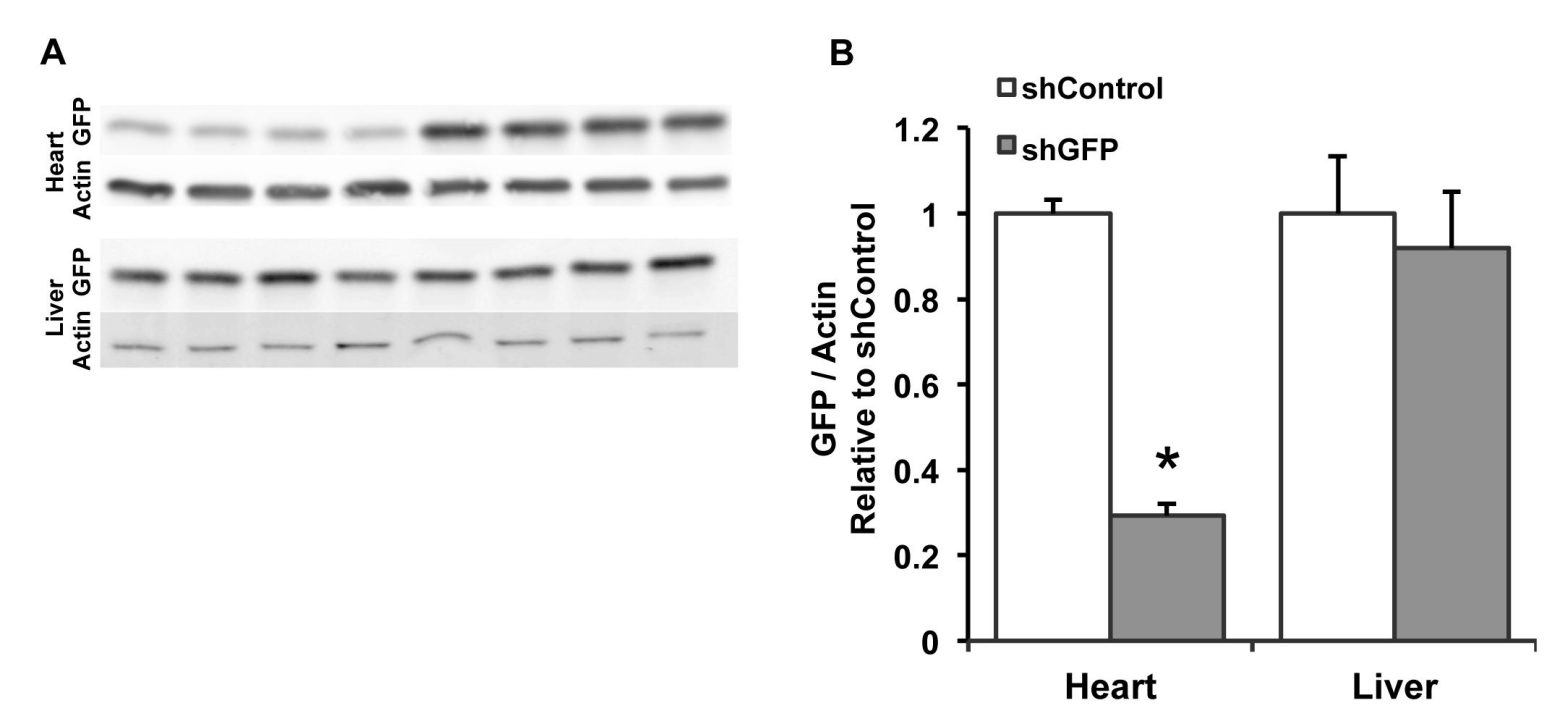
GFP protein in heart and liver. (A) Western blots of GFP in heart and liver tissues, with actin used as a loading control. The first four bands in each sample are from the shGFP group, while the second four bands are from the shControl group. (B) Quantitative analysis of the Western blots shows that actin-normalized GFP expression was reduced by 71% in the shGFP-treated group compared to the shControl group in heart (n=4/group; p<0.0001), but there was no difference in GFP expression between groups in the liver.

Next, Western blot analysis was performed on protein isolated from heart and liver (Figure 8). Actin-normalized GFP expression was reduced by 71% in the shGFP-treated group compared to the shControl group in heart (n=4/group; p<0.0001), but there was no difference in GFP expression between groups in the liver. These results corroborate the data from fluorescence microscopy of tissue sections and mRNA analysis, demonstrating that knockdown of GFP in the heart is highly efficient with no significant reduction of GFP expression in the liver.

**Figure 9:**
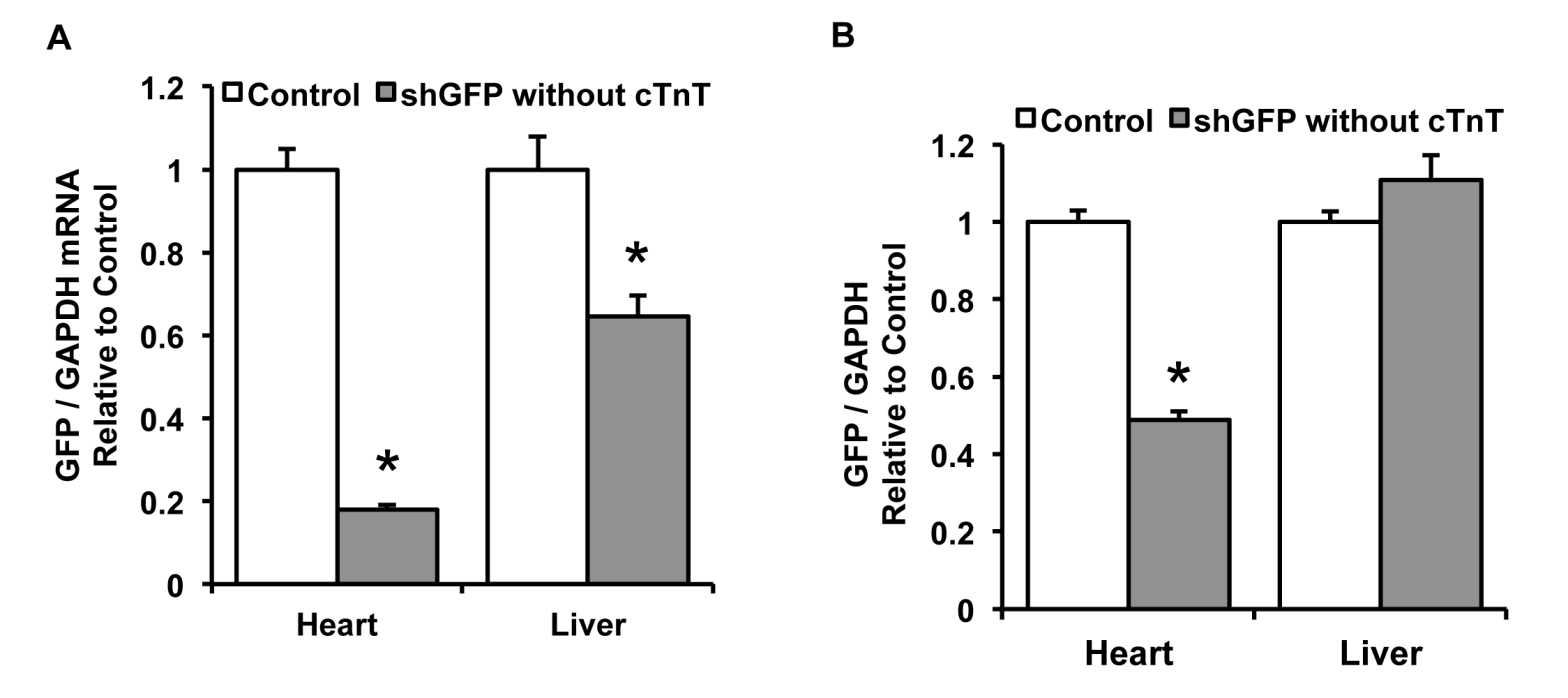
GFP mRNA and protein in heart and liver after treatment with the pAUSiGrLuc vector. To address the question of whether the cardiac troponin T promoter in the pAUSiGTL vector might contribute to cardiac-selective knockdown, we tested the pAUSiGrLuc vector which contained luciferase without the cTnT promoter. (A) GAPDH-normalized mRNA shows an 82% reduction compared to an untreated control group in the heart (n=5-8/group, p<0.0001) and a 36% reduction in GFP mRNA compared to controls in the liver (n=5-8/group, p<0.01). (B) Western blot analysis showed that GAPDH-normalized GFP expression was reduced by 51% in the shGFP-treated group compared to controls in heart (n=4/group, p<0.0001), with no difference in GFP expression between groups in the liver.

Finally, to address the question of whether the cardiac troponin T promoter in the pAUSiGTL vector might contribute to the cardiac-selective knockdown, we tested the pAUSiGrLuc vector which contained a luciferase stuffer in reverse orientation without the cTnT promoter. Ten day old mice treated with this vector showed a similar pattern of knockdown, with an 82% reduction in GAPDH-normalized GFP mRNA compared to an untreated control group in the heart (n=5-8/group, p<0.0001) and a 36% reduction in GFP mRNA compared to controls in the liver (n=5-8/group, p<0.01) (Figure 9A). Western blot analysis showed that GAPDH-normalized GFP expression was reduced by 51% in the shGFP-treated group compared to controls in heart (n=4/group, p<0.0001), a reduction in knockdown compared to the previous experiment that is likely due to an approximately 20% decrease in viral dose relative to mouse body mass at the time of injection. As before, there was no difference in GFP expression between groups in the liver (Figure 9B).

## Discussion

RNA interference-based gene therapy is an area of intense research with a great deal of potential for basic science investigation and clinical application. However, its implementation faces several challenges beyond those of traditional gene therapy. One is that gene knockdown is generally less predictable than gene overexpression. With a functioning promoter and cDNA, it is generally straightforward to overexpress any gene of interest. In contrast, it can often be challenging to identify the optimal region of a transcript for the most specific and efficiently targeted knockdown. Another challenge is attaining tissue-specific expression. mRNA transcripts are produced by RNA polymerase II promoters, which can be specific to a tissue of interest. Achieving adequate expression of interfering RNAs, however, is more efficiently achieved with RNA polymerase III promoters. This can make the task of targeting shRNA expression to a particular tissue quite challenging.

Here, we report highly efficient knockdown of GFP in the heart but not in the liver after systemic injection of AAV9 carrying a single-stranded genome. To our knowledge, this is the first study to examine knockdown in heart and liver after systemic delivery of AAV9. This is an important comparison since AAV9 has been shown to traffic to these two tissues more than any other [15,16], and previous reports have suggested the possibility of liver damage from shRNA expression [9]. We also describe the use of the luciferase reporter to confirm AAV9-mediated gene delivery prior to tissue level analysis of knockdown. Other labs have reported upon the use of AAV9 to knockdown genes in the heart [6,7,8], but the reporter system we employed here with ubc-GFP mice provides new insights into the tissue-wide distribution of knockdown (e.g., Figure 5B) that have not previously been described. The cell-to-cell variability in GFP signal intensity, notable particularly in the dosing study (Figure 3), is complementary to results our laboratory has previously reported in mice injected with AAV9 carrying GFP under control of the cardiac troponin T promoter [4]. In addition, because AAV9 has not previously been shown to effectively target non-cardiomyocytes in the heart, the 71% reduction of GFP in heart tissue may largely be mediated within cardiomyocytes. Since cardiomyocytes account for approximately 56% of cells in the heart [17], it is possible that this approach is achieving cardiomyocyte knockdown of greater than 71%.

To determine whether the cardiac-selective knockdown reported here was related to the downstream cardiac troponin T promoter used to drive luciferase expression, we tested an additional vector without the cTnT promoter. Mice treated with this vector also showed robust cardiac knockdown with no liver knockdown of GFP (Figure 9), comparable to mice treated with AAV that contained the cTnT promoter (Figure 8). Therefore it is likely that another mechanism is responsible for the lack of knockdown in the liver. A study by Mayra et al. described AAV9-mediated knockdown in cardiac and skeletal muscle after intraperitoneal delivery into neonatal mice, with no apparent shRNA production in the liver [18]. This preference for muscle over liver could be related to significant decreases in viral genomes per liver cell reported within 1-2 weeks after AAV8 delivery to neonatal mice, likely due to rapid division of hepatocytes in neonates [19,20]. Similarly, Wang et al. detected viral genomes in the heart but not in the liver 2 months after intraperitoneal administration of AAV8 to neonates [21]. In the current study, we delivered AAV9 to 8 day old mice via systemic injection and harvested tissues 7 weeks later. While we cannot rule out some dilution or degradation of viral genomes in the liver over that period of time, quantitative PCR showed 4.5-fold more AAV genomes in the liver than in the heart. Furthermore, this biodistribution of viral genomes is similar to what other studies have shown after systemic injection of adult mice [15,22].

A recent study by Lovric et al. suggests that increased AAV transduction in the heart may be due to terminal differentiation of cardiomyocytes followed by downregulation of the DNA-damage response MRN complex, which has been found to bind AAV genomes and may inhibit transduction through transcriptional silencing [23,24,25]. By contrast, hepatocytes retain the capacity for mitosis and show consistently higher expression of MRN proteins than the heart. Lovric et al. showed that co-administration of AAV with siRNAs targeting MRN expression dramatically increased AAV transduction in the liver without affecting the biodistribution of viral genomes. Thus the differential activity of MRN complexes in heart versus liver may also contribute to our finding of increased knockdown in the heart despite a greater number of viral genomes in the liver. Similarly, a previous comparison of AAV-mediated lacZ expression in cardiac and liver tissues by Inagaki et al. showed that to reach 83% transduction efficiency in the liver required a 10-fold higher dose of AAV9 than that needed to achieve similar efficiency in the heart [16]. Taken together, these studies suggest that the contrast in tissue knockdown described here may be due in part to the greater transduction efficiency of AAV9 in heart versus liver.

This study demonstrates that single-stranded AAV9 vectors expressing shRNA can be used to achieve highly efficient cardiac-selective knockdown of GFP expression 7 weeks after systemic administration to 8 day old mice, with no change in liver expression despite a heavy accumulation of AAV genomes in the liver. While the exact mechanism(s) responsible for this remarkable contrast in cardiac versus liver transduction remain the subject of ongoing investigation, our results suggest that the use of cardiac-specific promoters for gene knockdown may be unnecessary when using single-stranded AAV9 due to its intrinsic transduction efficiency in cardiomyocytes.

## References

[1] Wu Z, Asokan A, Samulski RJ (2006) Adeno-associated Virus Serotypes: Vector Toolkit for Human Gene Therapy. Molecular Therapy 14: 316–327. doi:10.1016/j.ymthe.2006.05.009. PubMed: 16824801. 16824801 10.1016/j.ymthe.2006.05.009

[2] Prasad K-MR, Xu Y, Yang Z, Toufektsian M-C, Berr SS et al. (2007) Topoisomerase Inhibition Accelerates Gene Expression after Adeno-associated Virus-mediated Gene Transfer to the Mammalian Heart. Molecular Therapy 15: 764–771. PubMed: 17299410. 10.1038/sj.mt.6300071 17299410

[3] Aikawa R, Huggins GS, Snyder RO (2002) Cardiomyocyte-specific Gene Expression Following Recombinant Adeno-associated Viral Vector Transduction. J Biol Chem 277: 18979–18985. doi:10.1074/jbc.M201257200. PubMed: 11889137. 11889137 10.1074/jbc.M201257200

[4] Prasad KMR, Xu Y, Yang Z, Acton ST, French BA (2011) Robust cardiomyocyte-specific gene expression following systemic injection of AAV: in vivo gene delivery follows a Poisson distribution. Gene Therapy 18: 43–52. doi:10.1038/gt.2010.105. PubMed: 20703310. 20703310 10.1038/gt.2010.105 PMC2988989

[5] Ku G, McManus MT (2008) Behind the Scenes of a Small RNA Gene-Silencing Pathway. Hum Gene Ther 19: 17–26. doi:10.1089/hum.2007.1226. PubMed: 18211225. 18211225 10.1089/hum.2007.1226

[6] Fechner H, Sipo I, Westermann D, Pinkert S, Wang X et al. (2008) Cardiac-targeted RNA interference mediated by an AAV9 vector improves cardiac function in coxsackievirus B3 cardiomyopathy. J Mol Med 86: 987–997. doi:10.1007/s00109-008-0363-x. PubMed: 18548221. 18548221 10.1007/s00109-008-0363-x

[7] Park CS, Cha H, Kwon EJ, Jeong D, Hajjar RJ et al. (2012) AAV-Mediated Knock-Down of HRC Exacerbates Transverse Aorta Constriction-Induced Heart Failure. PLOS ONE 7: e43282. doi:10.1371/journal.pone.0043282. PubMed: 22952658. 22952658 10.1371/journal.pone.0043282 PMC3429470

[8] Miyazaki Y, Ikeda Y, Shiraishi K, Fujimoto SN, Aoyama H et al. (2012) Heart Failure-Inducible Gene Therapy Targeting Protein Phosphatase 1 Prevents Progressive Left Ventricular Remodeling. PLOS ONE 7: e35875. doi:10.1371/journal.pone.0035875. PubMed: 22558250. 22558250 10.1371/journal.pone.0035875 PMC3338799

[9] Grimm D, Streetz KL, Jopling CL, Storm TA, Pandey K et al. (2006) Fatality in mice due to oversaturation of cellular microRNA/short hairpin RNA pathways. Nature 441: 537–541. doi:10.1038/nature04791. PubMed: 16724069. 16724069 10.1038/nature04791

[10] Schaefer BC, Schaefer ML, Kappler JW, Marrack P, Kedl RM (2001) Observation of Antigen-Dependent CD8+ T-Cell/ Dendritic Cell Interactions in Vivo. Cell Immunol 214: 110–122. doi:10.1006/cimm.2001.1895. PubMed: 12088410. 12088410 10.1006/cimm.2001.1895

[11] Tiscornia G, Singer O, Ikawa M, Verma IM (2003) A general method for gene knockdown in mice by using lentiviral vectors expressing small interfering RNA. Proc Natl Acad Sci USA 100: 1844–1848. doi:10.1073/pnas.0437912100. PubMed: 12552109. 12552109 10.1073/pnas.0437912100 PMC149921

[12] Graham FL, Smiley J, Russell WC, Nairn R (1977) Characteristics of a Human Cell Line Transformed by DNA from Human Adenovirus Type 5. J Gen Virol 36: 59–72. doi:10.1099/0022-1317-36-1-59. PubMed: 886304. 886304 10.1099/0022-1317-36-1-59

[13] Wu JC, Inubushi M, Sundaresan G, Schelbert HR, Gambhir SS (2002) Optical Imaging of Cardiac Reporter Gene Expression in Living Rats. Circulation 105: 1631–1634. doi:10.1161/01.CIR.0000014984.95520.AD. PubMed: 11940538. 11940538 10.1161/01.cir.0000014984.95520.ad

[14] Konkalmatt PR, Wang F, Piras BA, Xu Y, O’Connor DM et al. (2012) Adeno-associated virus serotype 9 administered systemically after reperfusion preferentially targets cardiomyocytes in the infarct border zone with pharmacodynamics suitable for the attenuation of left ventricular remodeling. J Gene Med 14: 609–620. doi:10.1002/jgm.2673. PubMed: 23065925. 23065925 10.1002/jgm.2673 PMC3729029

[15] Zincarelli C, Soltys S, Rengo G, Rabinowitz JE (2008) Analysis of AAV Serotypes 1–9 Mediated Gene Expression and Tropism in Mice After Systemic Injection. Molecular Therapy 16: 1073–1080. doi:10.1038/mt.2008.76. PubMed: 18414476. 18414476 10.1038/mt.2008.76

[16] Inagaki K, Fuess S, Storm TA, Gibson GA, Mctiernan CF et al. (2006) Robust Systemic Transduction with AAV9 Vectors in Mice: Efficient Global Cardiac Gene Transfer Superior to That of AAV8. Molecular Therapy 14: 45–53. doi:10.1016/j.ymthe.2006.03.014. PubMed: 16713360. 16713360 10.1016/j.ymthe.2006.03.014 PMC1564441

[17] Banerjee I, Fuseler JW, Price RL, Borg TK, Baudino TA (2007) Determination of cell types and numbers during cardiac development in the neonatal and adult rat and mouse. Am J Physiol Heart Circ Physiol 293: H1883–H1891. doi:10.1152/ajpheart.00514.2007. PubMed: 17604329. 17604329 10.1152/ajpheart.00514.2007

[18] Mayra A, Tomimitsu H, Kubodera T, Kobayashi M, Piao W et al. (2011) Intraperitoneal AAV9-shRNA inhibits target expression in neonatal skeletal and cardiac muscles. Biochem Biophys Res Commun 405: 204–209. doi:10.1016/j.bbrc.2011.01.009. PubMed: 21219850. 21219850 10.1016/j.bbrc.2011.01.009

[19] Cunningham SC, Dane AP, Spinoulas A, Alexander IE (2008) Gene Delivery to the Juvenile Mouse Liver Using AAV2/8 Vectors. Mol Ther 16: 1081–1088. doi:10.1038/mt.2008.72. PubMed: 18414478. 10.1038/mt.2008.72 28178471

[20] Flageul M, Aubert D, Pichard V, Nguyen TH, Nowrouzi A et al. (2009) Transient expression of genes delivered to newborn rat liver using recombinant adeno-associated virus 2/8 vectors. J Gene Med 11: 689–696. doi:10.1002/jgm.1343. PubMed: 19455564. 19455564 10.1002/jgm.1343

[21] Wang Z, Zhu T, Qiao C, Zhou L, Wang B et al. (2005) Adeno-associated virus serotype 8 efficiently delivers genes to muscle and heart. Nat Biotechnol 23: 321–328. doi:10.1038/nbt1073. PubMed: 15735640. 15735640 10.1038/nbt1073

[22] Shen S, Bryant KD, Sun J, Brown SM, Troupes A et al. (2012) Glycan Binding Avidity Determines the Systemic Fate of Adeno-Associated Virus Type 9. J Virol 86: 10408–10417. doi:10.1128/JVI.01155-12. PubMed: 22787229. 22787229 10.1128/JVI.01155-12 PMC3457279

[23] Lovric J, Mano M, Zentilin L, Eulalio A, Zacchigna S et al. (2012) Terminal Differentiation of Cardiac and Skeletal Myocytes Induces Permissivity to AAV Transduction by Relieving Inhibition Imposed by DNA Damage Response Proteins. Mol Ther 20: 2087–2097. doi:10.1038/mt.2012.144. PubMed: 22850678. 22850678 10.1038/mt.2012.144 PMC3493462

[24] Schwartz RA, Palacios JA, Cassell GD, Adam S, Giacca M et al. (2007) The Mre11/Rad50/Nbs1 Complex Limits Adeno-Associated Virus Transduction and Replication. J Virol 81: 12936–12945. doi:10.1128/JVI.01523-07. PubMed: 17898048. 17898048 10.1128/JVI.01523-07 PMC2169118

[25] Cervelli T, Palacios JA, Zentilin L, Mano M, Schwartz RA et al. (2008) Processing of recombinant AAV genomes occurs in specific nuclear structures that overlap with foci of DNA-damage-response proteins. J Cell Sci 121: 349–357. doi:10.1242/jcs.003632. PubMed: 18216333. 18216333 10.1242/jcs.003632

